# New fossils of Elateridae (Insecta, Coleoptera) from Early Cretaceous Jinju Formation (South Korea) with their implications to evolutionary diversity of extinct Protagrypninae

**DOI:** 10.1371/journal.pone.0225502

**Published:** 2019-12-11

**Authors:** Jae-Cheon Sohn, Gi Soo Nam, Sei-Woong Choi, Dong Ren

**Affiliations:** 1 Department of Science Education, Gongju National University of Education, Gongju, Chungnam, Republic of Korea; 2 Department of Environmental Education, Mokpo National University, Muan, Jeonnam, Republic of Korea; 3 Capital Normal University, Beijing, PR China; Museum National d’Histoire Naturelle, FRANCE

## Abstract

Two new genera and species of Elateridae, *Megalithomerus magohalmii* gen. et sp. nov. and *Koreagrypnus jinju* gen. et sp. nov., are described based on two pairs of fossils from the late Early Cretaceous Jinju Formation in Jinju City, South Korea. Both *Megalithomerus* and *Koreagrypnus* represent the youngest occurrences of an extinct elaterid subfamily, Protagrypninae. *Megalithomerus magohalmii* is the largest known fossil elaterid. These newly described elaterids provide a better understanding of the morphological diversity and occurrence of Protagrypninae through geologic time.

## Introduction

Elateridae, commonly known as click beetles, are the most speciose family in the superfamily Elaterioidea, which includes nearly 10,000 species worldwide [1]. Adult elateroids can generally be recognized by their elongate, narrow body, and their disproportionately large and freely articulating prothorax. The largest group within the superfamily, Elateridae differ from others in the number of connate ventrites and the shapes of antenna and prothorax [2]. The most significant characteristic of these beetles is their ability to jump into the air via a jack-knifing movement in which the prosternal process slides rapidly down a smooth track into the mesosternal cavity [3, 4]. A sudden flexing movement of their prothorax produces an audible clicking or snapping, from which their common name originates. Despite their variety of sizes and shapes, Elateridae possess the same morphology of a clicking device [5]. Adult elaterids feed on predominantly dead or live plant materials, but a few prey on soft-bodied insects [2]. Elaterid larvae, commonly known as wireworms, primarily live in decaying wood and litter within the soil and some cause economic damage to a large variety of crops [6].

Costa *et al*. [1] divided Elateridae into 19 subfamilies, based on a synthesis of previous phylogenetic hypotheses [7, 8]. These include several small groups, such as Cebrioninae, Diminae and Thylacosterninae, whose phylogenetic status remains unstable. This problem stems from a lack of morphological evidence clearly defining the subfamilies [9]. These problematic lineages led to weak support for the elaterid monophyly. Recent molecular phylogenetic studies [9–12] recovered Elateridae as monophyletic once the problematic subfamilies were excluded.

The fossil record of Elateridae comprises 271 named species and another 40 species that had not been fully named (updated from Kirejthuk & Ponomarenko [13]). All of these elaterid species were assigned to at least 12 subfamilies and 112 genera including 44 extant genera. The earliest fossils of reliably-identified Elateridae are known from the Early Jurassic [14], although there is a dubious record from the latest Triassic Apperley beds of the United Kingdom [15]. Numerous elaterid fossils have been reported from the Daohugou beds of Northeastern China (Middle Jurassic), the Karabastau Formation in Kazakhstan (Late Jurassic), the Yixian Formation in China (Early Cretaceous), Baltic amber from the Prussian Formation (Middle Eocene), and the Florissant Formation in the United States (Late Eocene).

The Jinju formation has yielded several insect fossils including Blattodea [16], Odonata [17, 18], Raphidioptera [19], Dermaptera [20], and Coleoptera [21] as well as microbial biohermal deposits rich in caddisfly cases [22]. However, many insect fossils from the formation remain unstudied. In the present article, we describe two fossil species of click beetles from the Jinju Formation. These represent the first fossil records of Elateridae from Korea. One of the species described in this study is the largest fossil of Elateridae, although there exist even larger species in the extant fauna.

## Material and methods

### Geological setting

The click-beetle fossils were discovered from the Jinju Formation located in the Jeongchon Section of Jinju City, Gyeongsangnamdo Province, South Korea (35°07'45"N, 128°06'02"E). This formation, which belongs to a lacustrine Cretaceous deposit, Gyeongsang Supergroup [23], consists of black shale, light gray arkose, and greenish gray sandy shale [24]. The age of this formation has been debated, with dates ranging from Hauterivian–Barremian [25–28] to Albian [29].

The Jinju Formation has yielded plants, ostracodes, bivalves, conchostacans, insects, spiders, stromatolites, dinosaur bones and tracks, and pterosaur teeth [21, 30]. Park *et al*. [31] reported the occurrence of *Archaeoniscus*, an isopod group living in shallow sea during the Jurassic–Early Cretaceous. Choi [25] examined palynofloras from the formation and suggested that an arid and warm climate was prevalent in the region during the Early Cretaceous.

### Examination

The type specimens have been deposited in the Paleontology collection of the Gongju National University of Education, Woongjinro 27, Gongju, Republic of Korea (GNUE). The specimens were examined using two types of dissecting microscopes (Nikon SMZ 800 and Leica EZ4) and photographed using a digital camera (Nikon D40) in an all-purpose light box or with an adapter connecting to a dissecting microscope (Leica MZ6). Terms of external morphology follow Lawrence *et al*. [32]. The measurement definitions follow Chang *et al*. [33] and these are:

- The body length [BL] from the apex of the mandible to the apex of the abdomen;
- The body width [BW] at the base of the elytra);
- The length and width of the pronotum [PNL & PNW] measured at the median line;
- The elytron length [EL] from the base to the apex;
- The elytron width [EW] between the two anterior corners.

#### Nomenclatural acts

The electronic edition of this article conforms to the requirements of the amended International Code of Zoological Nomenclature, and hence the new names contained herein are available under that Code from the electronic edition of this article. This published work and the nomenclatural acts it contains have been registered in ZooBank, the online registration system for the ICZN. The ZooBank LSIDs (Life Science Identifiers) can be resolved and the associated information viewed through any standard web browser by appending the LSID to the prefix "http://zoobank.org/". The LSID for this publication is: urn:lsid:zoobank.org:pub: B3AB9668-8CD2-4F52-84FB-B1FC32A4F5BA. The electronic edition of this work was published in a journal with an ISSN, and has been archived and is available from the following digital repositories: PubMed Central, LOCKSS.

## Systematic paleontology

Order Coleoptera Linnaeus, 1758

Family Elateridae Leach, 1815

Subfamily Protagrypninae Dolin, 1973

Tribe Protagrypnini Dolin, 1973

**Genus *Megalithomerus* Sohn et Nam, gen. nov**. urn:lsid:zoobank.org:act:674F50AC-FB39-4CAB-A899-33DA64E65B42.

**Type species**. *Megalithomerus magohalmii* Sohn et Nam, sp. nov., by monotypy.

**Diagnosis**. Among the Cretaceous Protoagrypninae, this genus is similar to *Paralithomerus* Chang, Zhang et Ren, 2008, but differs from the latter in having an elongate mesosternal cavity (subquadrate in *Paralithomerus*). *Megalithomerus* is also similar to two Middle-Jurassic genera, *Paraprotagrypnus* Chang, Zhao et Ren, 2009 and *Protagrypnus robustatus* Chang, Kirejtshuk, Ren et Shih, 2009, but differs from *Paraprotagrypnus* in having two pairs of grooves (one pair in *Paraprotagrypnus*) on the prosternum and the more elongate mesosternal cavity and from *Protagrypnus* in having an additional groove on the prosternum and an elongate mesoventrite cavity.

**Description**. This genus is characterized by a combination of the following characteristics: i) body length exceeding 40 mm; ii) postlateral margin of pronotal disc slightly curved before posterior carina; iii) posterior carina of pronotal disc with a deep groove ventrally; iv) prosternum with two pairs of shallow grooves; v) lateral margin of prosternum nearly straight; vi) mesosternal cavity elongate; vii) mesocoxal cavity surrounded by mesosternum, metasternum, and mesepisternum; viii) metacoxal plate not reaching lateral margin of basal abdominal ventrite; and ix) clear striae on elytron absent.

**Etymology**. The generic name is derived from a combination of the Greek prefix ‘*megas*’, meaning “large”, and the existing genus *Lithomerus*, which refers to its resemblance to *Lithomerus* and large size.

**Remarks**. This genus undoubtedly belongs to Elateridae given its prothorax, with backward-pointing carina on the posterior corners; the presence of a prosternal process and a mesoventrial cavity; and well developed metacoxal plates. Exclusion of *Megalithomerus* from Cerophytidae and Throscidae is substantiated by the lacks of the laterally-produced posterior angles of pronotum and the grooves on the metaventrite, respectively. It also differs from Eucnemidae in the presence of three connate ventrites. Dolin [34] defined Protoagrypninae based on three characteristics. *Megalithomerus* exhibit two of those: the presence of additional furrows on the prosternum and the presence of a transverse suture on the mesoventrite. Only unpolarized characters associate *Megalithomerus* with Protoagrypnini and they include the presence of the pronotosternal sutures apparently associated with sulciform grooves; the procoxae opened posteriorly; and the mesepisternum with a transverse suture.

**Fig 1:**
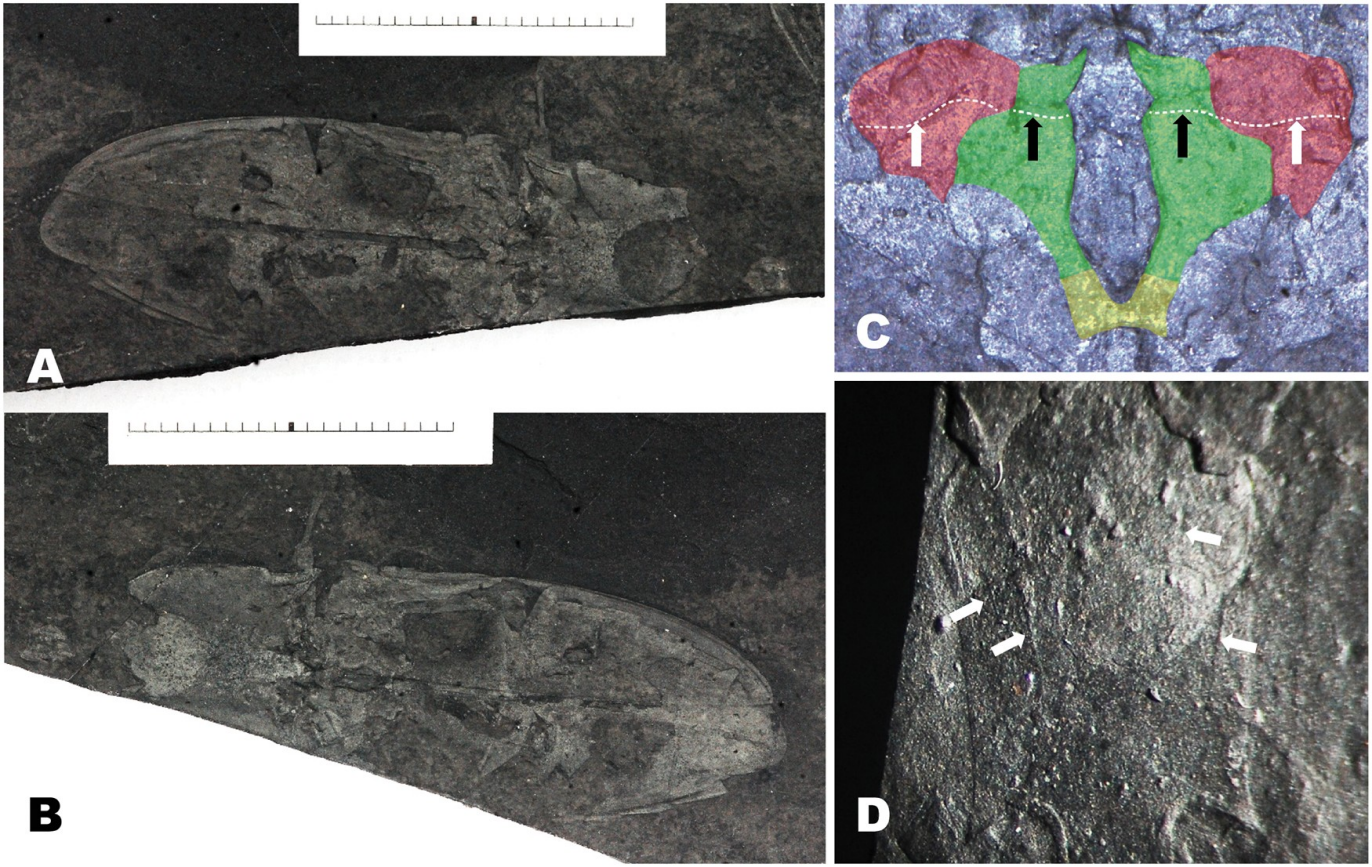
*Megalithomerus magohalmii* Sohn et Nam, gen. et sp. nov. (A) Holotype GNUE-I-2013001, habitus, units in measure = mm. (B) Counterpart of holotype GNUE-I-2013001c, habitus, units in measure = mm. (C) Close-up of mesoventrite (green), mesepisternum (red), and metaventrite (yellow), black arrows indicating transverse sutures on mesoventrite, white arrows indicating transverse sutures on mesepisternum. (D) Close-up of prosternum, white arrows indicating two pairs of grooves.

**Fig 2:**
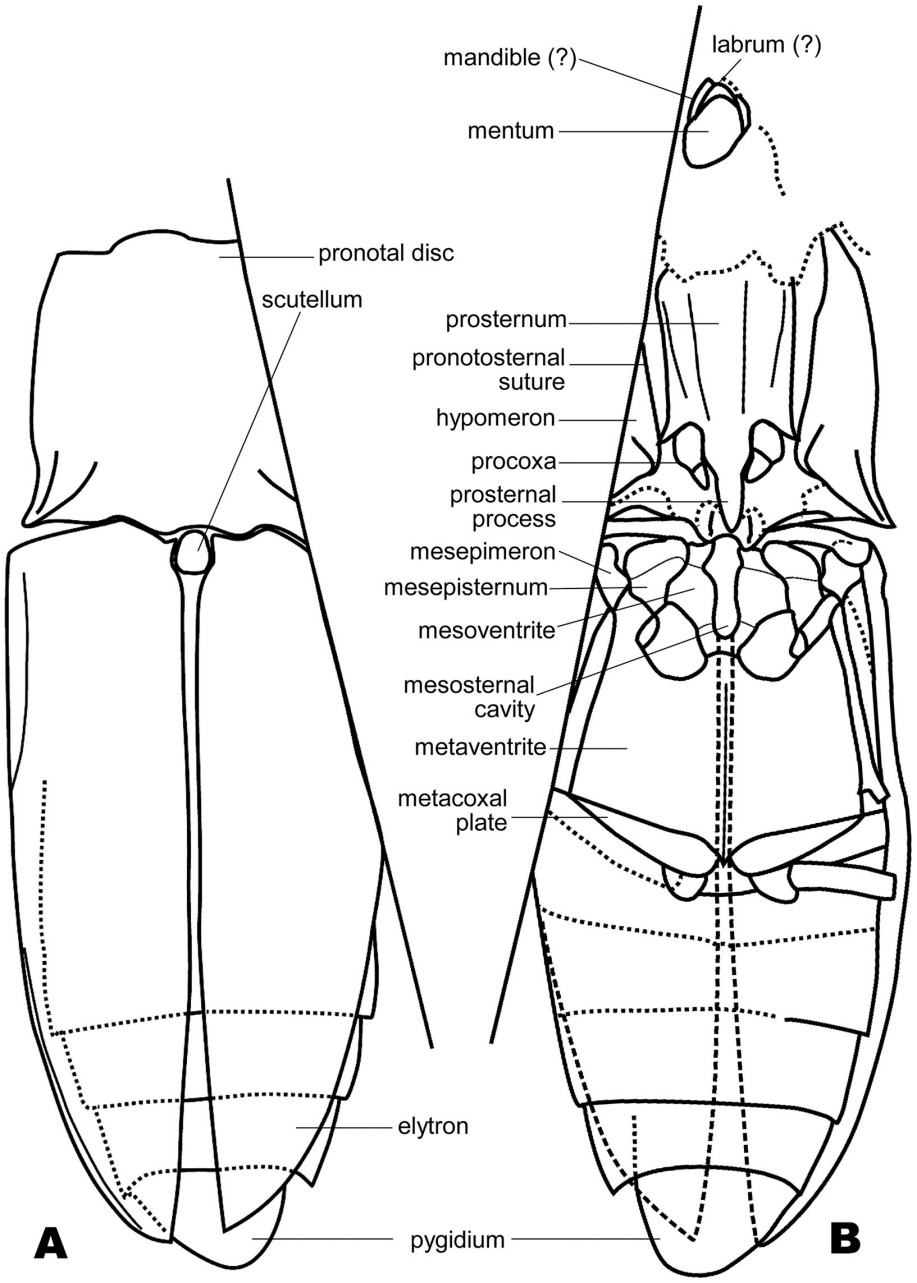
Line drawings of *Megalithomerus magohalmii* Sohn et Nam, gen. et sp. nov. (A) Dorsal view, GNUE-I-2013001. (B) Ventral view, GNUE-I-2013001c.

***Megalithomerus magohalmii* Sohn et Nam, sp. nov. (Figs 1 and 2)** urn:lsid:zoobank.org:act:E7914A94-66A4-4047-95AA-AAD69A52538D.

**Type**. Holotype–GNUE-I-2013001 and GNUE-I-2013001c (original part and counterpart of one specimen).

**Diagnosis**. Same as generic diagnosis.

**Preservation**. Parts of head, including mandibles, labrum, and mentum are preserved. The anterior part of pronotum is concealed by rock matrix. A straight section obliquely cuts off parts of thorax and abdomen, but at least one side of these parts are well-preserved. The legs, except the coxal cavities, are missing, but the mesofemur, mesotibia and metafemur on one side are visible.

**Description**. Head–Mentum possibly subtriangular; mandible broadly arched, bidentate at apex. Prothorax–Pronotum as wide as its length, 1/2 as long as elytron; disc broadly, weakly convex on medial area; lateral margin broadly round; hind angle acuminate, with short carina producing posterolaterally, 1/5 as long as disc; carina with a deep groove ventrally; posterior margin slightly concave medially and at lateral half. Prosternum (Fig 1d) rectangular except prosternal process, slightly emarginated around procoxal cavities, with two pairs of shallow, longitudinal grooves laterally; pronotosternal suture double, widely open anteriorly; prosternal process 1/2 as long as lateral margin of prosternum, bullet-shaped, with triangular apical area 1/2 of its length. Procoxal cavities elliptical, separated, open posteriorly. Pterothorax–Scutellum subtriangular, narrowly round anteriorly. Mesoventrite (Fig 1c) nearly hexagonal, with transverse sutures at anterior 2/5; mesosternal cavity digitate, narrowed basally. Mesepisternum subtriangular, with obliquely-transverse suture at anterior 1/3. Mesepimeron subquadrate. Mesovetrite and metaventrite separated by short suture. Mesocoxa open to mesepimeron. Mesotibia 3 times longer than mesofemur, with short tooth subapically. Metaventrite nearly trapezoidal, flat, with longitudinal suture medially; metepisternum very long; metacoxal plate airfoil-shaped, tapered laterally. Elytra as wide as pronotum, slightly convex along lateral margin, irregularly punctate, with no distinct striae; ratio of elytral length to greatest elytral width 2.14; ratio of elytral length to pronotal length 2.09; elytral apices almost meeting along suture except distal 1/3. Abdomen–Basal ventrite broadened to middle, slightly concave postmedially. Five sternite visible, gradually narrowed from first to forth visible sternite; pygidium narrowly round apically.

**Measurements (mm)**. BL: 47.8; BW: 13.8; PNL: ca. 12; PNW: ca. 12; EL: 27; EW: 6.8.

**Locality and horizon**. Jeongchon Section, Jeongchon-myeon, Jinju-si, Gyeongsangnamdo Province, South Korea; Jinju Formation; Albian, late Early Cretaceous.

**Etymology**. This large species was named after a giant goddess ‘magohalmi’ in a Korean folklore.

**Remarks**. This species is the largest, described elaterid fossil to date, although a few extant elaterids exceed the body size of *M*. *magohalmii*.

**Genus *Koreagrypnus* Sohn et Nam, gen. nov**. urn:lsid:zoobank.org:act:A4DA0A05-CBF6-4A90-8314-DB53F3D42DD9.

**Type species**. *Koreagrypnus jinju* Sohn et Nam, sp. nov., by monotypy.

**Diagnosis**. This genus is similar to *Paralithomerus* Chang, Zhang et Ren, 2008 from the Early Cretaceous Yixian Formation in the lack of a medial plate on the prosternum, but differs from the latter in having the smaller anterior opening of the pronotosternal sutures and the metacoxal plate reaching the lateral margin of the basal abdominal ventrite, and lacking the distinct striae on the elytron. It is also similar to a Late Jurassic genus, *Lithomerus* Dolin, 1980 in the shape of the metaventrite, but differs from the latter in having smaller eyes and a depressed area on the hypomeron.

**Description**. This genus is characterized by a combination of the following characteristics: i) eyes as long as antennal scape; ii) pronotum quadrate; iii); prosternum without medial plate; iv) hypomeron with a broadly depressed area along pronotosternal suture; v) mesoventrite cavity elongate; vi) posterior margin of metaventrite longer than its length; and vii) distinct striae on elytron absent.

**Etymology**. The generic name is derived from the extant elaterid genus *Agrypnus* combined with ‘Korea’, the country where this fossil was discovered.

**Remarks**. This genus is associated with Elateridae, based on three shared characteristics: a prothorax with backward-pointing carina on the posterior corners; the presence of a prosternal process and a mesoventrial cavity; and well developed metacoxal plates. *Koreagrypnus* is excluded from Cerophytidae, Throscidae, and Eucnemidae in the presence of posterolaterally-produced posterior angles of pronotum, the lack of the grooves on the metaventrite, and the presence of well-developed labrum on the head, respectively. Within Elateridae, *Koreagrypnus* can be assigned to Protoagrypninae based on the presence of a transverse suture on the mesoventrite, one of the characters defining the subfamily by Dolin [34]. A combination of three unpolarized characters helps *Koreagrypnus* assign to Protoagrypnini and these characters include the pronotosternal sutures apparently associated with sulciform grooves; the procoxae opened posteriorly; and the mesepisternum with a transverse suture.

**Fig 3:**
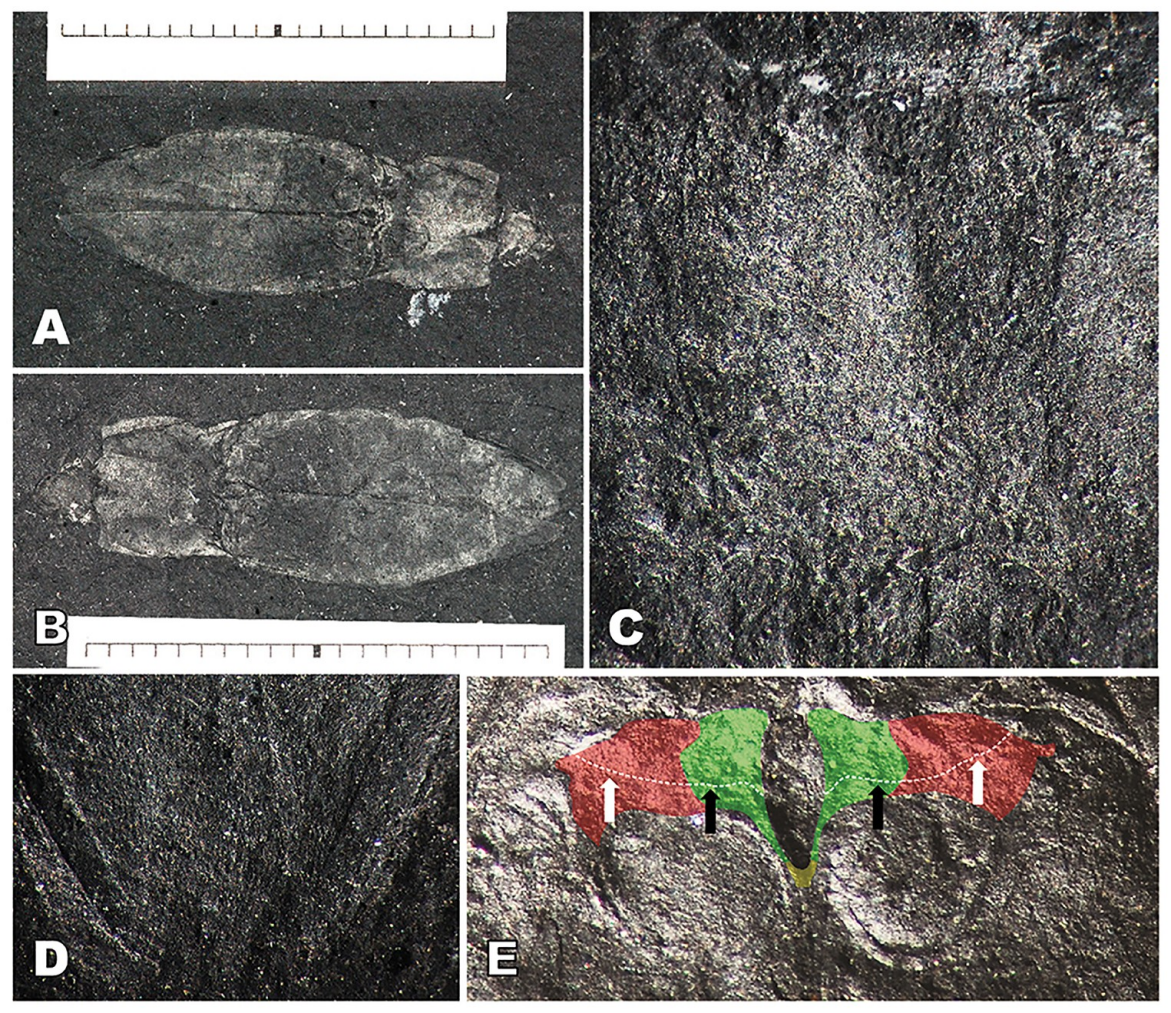
*Koreagrypnus jinju* Sohn et Nam, gen. et sp. nov. (A) Holotype GNUE-I-2013002, habitus, units in measure = mm. (B) Counterpart of holotype GNUE-I-2013002c, habitus, units in measure = mm. (C) Close-up of prosternum showing irregular punctures on surface. (D) Close-up of pygidium showing scobinated surface; **e**, close-up mesoventrite (green), mesepisternum (red), and metaventrite (yellow), black arrows indicating transverse sutures on mesoventrite, white arrows indicating transverse sutures on mesepisternum.

**Fig 4:**
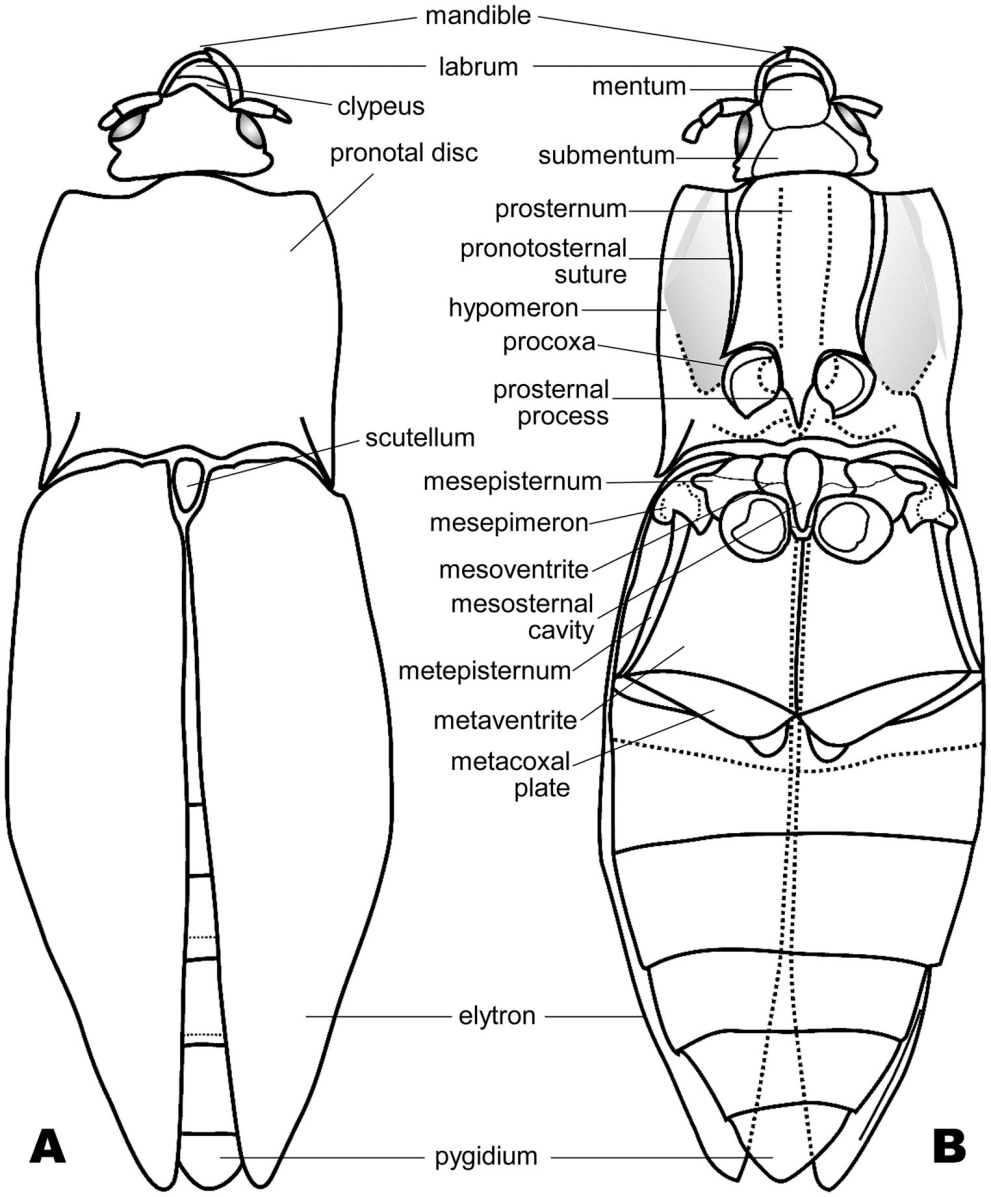
Line drawings of *Koreagrypnus jinju* Sohn et Nam, gen. et sp. nov. (A) Dorsal view, GNUE-I-2013002. (B) Ventral view, GNUE-I-2013002c.

***Koreagrypnus jinju* Sohn et Nam, sp. nov. (Figs 3 and 4)** urn:lsid:zoobank.org:act:4AE92074-5222-4346-84C2-A29CA478A406.

**Type**. Holotype–GNUE-I-2013002 and GNUE-I-2013002c (original part and counterpart of one specimen).

**Diagnosis**. Same as generic diagnosis.

**Preservation**. The entire head, parts of antennae, entire thorax and abdomen except legs, and coxal cavities are preserved.

**Description**. Head–Vertex semicircular, irregularly punctate; frons subtriangular; clypeus straight distally; labrum semi-elliptical; mentum subquadrate; mandible arched, bidentate at apex. Antenna possibly filiform; scape 2× longer than pedicel; 3^rd^ antennomere as long as pedicel. Prothorax–Pronotum almost as wide as its length, 2.6× shorter than elytron; disc slightly convex longitudinally, slightly depressed anteromedially, irregularly punctate; anterior margin convex medially; lateral margin broadly round; hind angle acuminate, with carina producing posteriorly, 1/4 as long as disc; posterior margin slightly bi-sinuous medially. Prosternum (Fig 3c) rectangular except prosternal process, broadly round anteriorly, slightly emarginated around procoxal cavities; pronotosternal suture double, gradually broadened to anterior opening; prosternal process 1/2 as long as lateral margin of prosternum, bullet-shaped, with triangular apical area 1/3 of its length. Hypomeron broadly depressed along pronotosternal suture, with oblique ridge anteriorly. Procoxal cavities subquadrate, slightly emarginated on outer margin, separated, broadly open. Pterothorax–Scutellum obovate. Mesoventrite (Fig 3e) subrectangular, concave laterally, convex anteriorly, with suture at middle; mesosternal cavity oval in anterior half, long-subtriangular in posterior half. Mesepisternum protruding into mesepimeron at middle, with suture at anterior 1/3. Mesepimeron overall triangular. Mesovetrite and metaventrite separated by short suture transversing mesoventrite cavity. Metaventrite trapezoidal, flat, with longitudinal suture medially; metepisternum as long as metaventrite; metacoxal plate blade-shaped, reaching lateral margin of basal abdominal ventrite. Metacoxal cavities close to each other. Elytra wider than pronotum at middle, irregularly punctate, with no distinct striae; lateral margin broadly round at middle; ratio of elytral length to greatest elytral width 1.99; ratio of elytral length to pronotal length 2.64; elytral apices meeting almost entirely. Abdomen–Basal ventrite nearly parallel to metacoxal plate, concave postmedially. Five sternites visible, gradually narrowed from first to forth visible sternite, scobinate (Fig 3d); pygidium subtriangular.

**Measurements (mm)**. BL: 23.1; BW: 6.5; PNL: 5.2; PNW: 6.1, EL: 14.8; EW: 3.0.

**Locality and horizon**. Jeongchon Section, Jeongchon-myeon, Jinju-si, Gyeongsangnamdo Province, South Korea; Jinju Formation; Albian, late Early Cretaceous.

**Etymology**. This species is named after Jinju, the city where the type specimens were discovered.

## Discussion

Protagrypninae is an extinct subfamily of Elateridae that includes 33 genera and 103 species [13]. This group was once treated as a tribe of Agrypninae [35] or Pyrophorinae [7] but the majority of other researchers [34, 36–38] regarded it as a separate subfamily. Protagrypninae exhibits two unique characteristics, the presence of a central plate on the prosternum and a transverse suture on the mesoventrite. Our assignments of two fossil species to Protagrypninae were based on the presence of those two characteristics. Chang *et al*. [36] suggested that the prosternum with a central plate is a primitive characteristic of Elateridae, but their suggestion needs to be tested by rigorous phylogenetic analyses.

The protagrypnine click beetles seemed to originate in the Early Jurassic and the lineage was present until the late Early Cretaceous [37]. The youngest fossils of the lineage come from the Laiyang Formation in China, not more than 130 million years ago [39]. Alexeev [38] described a new tribe of Protagrypninae, Pollostelasterini from the Zaza Formation of Russia whose age was suggested as Valanginian–Hauterivian [40]. In their review, Kirejtschuk & Azar [41] mentioned a possible protagrypnine inclusion in Lebanese amber whose age was estimated as Barremian [42]. The Jinju Formation, where *Megalithomerus* and *Koreagrypnus* were found, is about the same age or somewhat younger than the fore-mentioned sediments, although its exact age within the Early Cretaceous is debatable. Of the protagrypnine tribes, only Protagrypnini survived through the Jurassic to Cretaceous boundary interval. *Megalithomerus* and *Koreagrypnus* may represent the thriving, if not last, survivors of Protagrypnini.

Chang *et al*. [43] proposed that the several million-year-interval of the Late Jurassic to Early Cretaceous was a crucial time in the evolution of click beetles. Indeed, the fossils from the Yixian Formation in China display a transitional overlap of extinct and extant lineages of Elateridae during that time. No fossils of Protagrypninae have been found from the Late Cretaceous or younger fossil beds. The Protagrypninae were likely waning and finally became extinct in the Late Cretaceous, despite angiosperm radiation favoring the diversification of many herbivorous insects [44]. This interpretation, however, should be taken with caution given the poor fossil record of insects in the Late Cretaceous.

*Megalithomerus magohalmii* from the Early Cretaceous Jinju Formation is 47.8 mm in body length, representing the largest documented elaterid fossil. This beetle exhibits a typical clicking device in Elateroidea. Given its large body size, it might not be a high jumper. It is known that the height of jumps in extant elaterids reaches the highest levels for individuals with about 15 mm of body length, but decreases in height proportionally as the jumpers get smaller or larger than 15 mm [45]. This means that there is likely a size limit for saltatorial behavior in click beetles. The body length of *Megalithomerus magohalmii* might not reach the limit, since it was still far less than that of the largest jumpable, extant elaterid, *Oxynopterus auduoin* Hope.

## Conclusions

Two new species of protagrypnine elaterids were discovered from the Early Cretaceous Jinju Formation in South Korea. These beetles represent the youngest occurrence of Protagrypninae and show that the subfamily was lasting for a period of the Jurassic to the Early Cretaceous. One of those, *Megalithomerus magohalmii* is the largest fossil species of Elateridae.

## Abbreviations

BL: body length
BW: body width
DJSH: Daejeon Science High School for the Gifted
EL: elytron length
EW: elytron width
Ma: million years ago
PNL: pronotum length
PNW: pronotum width
